# Retraction: Effects of microRNA-211 on proliferation and apoptosis of lens epithelial cells by targeting SIRT1 gene in diabetic cataract mice

**DOI:** 10.1042/BSR-2017-0695_RET

**Published:** 2024-11-21

**Keywords:** Apoptosis, Diabetic cataract, Lens epithelial cells, microRNA-211, Proliferation, SIRTI

This article “Effects of microRNA-211 on proliferation and apoptosis of lens epithelial cells by targeting SIRT1 gene in diabetic cataract mice” DOI: 10.1042/BSR20170695 is being retracted from *Bioscience Reports* at the request of the Editor-in-Chief and the Editorial Board. This follows receipt of a notification from a reader, alerting the Editorial Board to parts of the flow cytometry scatter graphs in Figure 9A, which seem to appear in two subsequent papers: Figure 6D from Li et al, 2020 (DOI: 10.2147/ott.s222375), and Figure 3A from Sang et al, 2018 (DOI: 10.2147/ott.s169947).

The Editorial Office also has concerns regarding potential signs of digital splicing in Figure 3B Bax and p53 panels, and Figure 6A SIRT1, Bcl-2, and p53 panels. The authors have been contacted with regards to the retraction and have not responded to the Journal's queries or the concerns raised. Given the extent of the issues raised, the Editorial Board stand by the decision to retract the article.

